# Synthesis and Characterization of Cellulose Acetate—HBA/Poly Sulfone Blend for Water Treatment Applications

**DOI:** 10.3390/membranes15020038

**Published:** 2025-01-26

**Authors:** Lamai Alsulaiman, Abir S. Abdel-Naby, Salha Alharthi, Bushra ALabdullatif, Abeer Al-Dossary, Wafa Al-Mughrabi, Yanallah Alqarni

**Affiliations:** Chemistry Department, College of Science, Imam Abdulrahman Bin Faisal University, P.O. Box 1982, Dammam 31441, Saudi Arabia; 2220700031@iau.edu.sa (L.A.); snalharthi@iau.edu.sa (S.A.); 2210700017@iau.edu.sa (B.A.); aodossary@iau.edu.sa (A.A.-D.); walmagribi@iau.edu.sa (W.A.-M.); yaalqarni@iau.edu.sa (Y.A.)

**Keywords:** polysulfone, cellulose acetate, salt rejection, polymer blend, membranes

## Abstract

Cellulose acetate (CA) was chemically modified with p–hydrazinobenzoic acid (HBA) for the fabrication of a CA–HBA polymeric membrane. The CA–HBA was characterized using NMR, UV-Vis, and EDX/SEM techniques. CA–HBA exhibited high hydrophilicity, as it included carboxylic groups as well as the hydroxyl group of the CA glycosidic ring. The HBA moieties increased the hydrophilicity and the number of active sites inside the CA polymeric matrix, but they did not improve the thermal stability of the polymer, as shown by the thermogravimetry (TGA). Polysulfone (PSF) was blended with CA-HBA in various compositions to produce highly thermal and effective membranes for water treatment applications. The fabricated membranes (CA–HBA/PSF) (5:95) (10:90) (15:85) were found to exhibit high thermal stabilities. The CA–HBA/PSF 15:85 membrane exhibited the highest efficiency towards the removal of Cu (II) ions, while the 5:95 membrane exhibited the highest salt rejection (89%).

## 1. Introduction

The rapid development of different industrial processes, such as those used in the oil, petrochemical, textile, food, and metal industries, has led to the release of pollutants that deteriorate water quality and pose threats to health and ecological systems [1]. Sustainable industrial wastewater management has attracted more attention to the third global risk, which is water scarcity [2].

Heavy-metal-contaminated water has emerged as one of the most urgent global environmental problems due to the non-biodegradability, toxicity, carcinogenicity, and biological accumulation properties of heavy metals [3]. Residential or occupational exposure to an excess amount of heavy metals may result in DNA damage, hypertension, renal dysfunction, hallucinations, vertigo, or gastrointestinal distress [4,5]. Different strategies have been applied to remove metals from water sources, including adsorption, chemical precipitation, ion exchange, electrochemical deposition, and membrane filtration [6]. Among these strategies, membrane filtration has been proven as a prominent strategy for heavy metal removal due to its simplicity and low operational cost [7]. The technique for membrane fabrication is chosen according to the crystallinity of the polymeric matrix. For highly crystalline polymers, the electrospinning and centrifugal spinning strategies have gained considerable attention among all of the techniques used to produce membranes. Polysulfone is an amorphous polymer that exhibits high plasticity. To fabricate PSF membranes, phase inversion is the most suitable technique [8].

Polysulfone (PSF) is ranked as the most important polymer for membrane manufacturing because of its thermal stability, chemical resistance, mechanical strength, and optical transparency [9].

Despite the suitability of PSF for membrane fabrication, its high hydrophobicity, low salt-rejection capability, and high fouling tendency limit its application in various industrial fields for treating industrial wastewater [10]. Different methods have been integrated to improve the hydrophilicity of PSF and overcome the fouling problem, such as blending, coating, and grafting [11]. Other studies have been conducted to produce fouling-controlling membranes via blending PSF with inorganic nanoparticles. Recently, a PSF matrix was incorporated with Co^2+^ ion-doped ZnO nanoparticles, which resulted in a drop in the water contact angle from 82.7º (PSF) to 62º and an increase in the water flux to 48 L m^−2^ h^−1^, compared to 17.5 for neat PSF [12]. In a similar study, the hydrophilicity and salt rejection performance of PSF were improved by the addition of graphene oxide–zinc oxide (GO–ZnO) nanoparticles [13]. The utilization of TiO_2_ nanoparticles also improved the hydrophilicity, porosity, water permeability, and mechanical strength of PSF membranes [14]. Another common method used to impart a high fouling resistance into PSF membranes is polymer blending [15]. Several studies have reported the use of chitosan to enhance the hydrophilicity and anti-fouling properties of PSF due to chitosan’s hydrophilic characteristics [16,17,18]. Another polymeric material of particular interest to researchers for membrane fabrication is cellulose acetate (CA), which is one of the most essential cellulose derivatives. CA membranes offer several distinct benefits, including their construction from renewable resources, ease of manufacturing, affordability, and suitability for all pressure-driven membrane processes, such as microfiltration (MF), ultrafiltration (UF), nanofiltration (NF), and reverse osmosis (RO) [19,20]. Additionally, they are relatively hydrophilic, have a low tendency to foul, and are nontoxic. Pure CA membranes are known to exhibit a relatively low flux [19,21,22]. To address this limitation, researchers have proposed the incorporation of hydrophilic modifiers such as inorganic nanoparticles (NPs), including TiO_2_, Ag, Al_2_O_3_, ZnO, bentonite, zeolites, Fe_3_O_4_, and ZrO_2_. These modifications have been shown to improve the membrane flux and separation performance [23]. For example, CA/zeolite electrospun membranes have been studied for their effectiveness in oily wastewater separation [24,25].

CA has demonstrated several features, including high hydrophilicity and biodegradability and an ability to be functionalized to meet specific needs [20,26]. For example, nano-zerovalent iron (nZVI) and Al_2_O_3_ nanoparticles have been mixed with CA–PSF for the removal of methylene blue dye and Cu (II) metal ions. The generated CA–PSF/Al_2_O_3_ and CA–PSF/nZVI membranes exhibited a low water contact angle of 43º and 39º, respectively, and a high permeation flux of up to 85% and 88%, respectively [27]. Another study reported the suitability of CA blended with PSF in the presence of SiO2 for the removal of Cu (II), Fe (II), and Zn (II), with more than 90% efficiency [28]. 4–Hydrazinobenzoic acid (HBA) is the building block in the synthesis of many heterocyclic compounds and has distinct biological activities [29]. Considering the presence of electron donor groups such as amino (–NH2) and carboxylic (–COOH) groups in the structure of HBA, it was selected to facilitate the removal of heavy metals. Herein, CA was modified with HBA, followed by blending with PSF to afford a novel hybrid membrane for Cu (II) ion removal and water desalination applications.

## 2. Materials and Methods

### 2.1. Instruments

The characterization of compounds via nuclear magnetic resonance spectroscopy utilized a 400 MHz NMR device (Bruker Corporation, Bremen, Germany), employing DMSO-d_6_ as a solvent for sample dissolution. Scanning electron microscopy images were captured with a TESCAN VEGA3 (Brno, Czech Republic) scanning electron microscope, with a secondary electron (SE) detector, an energy-dispersive spectrometer detector, and electron backscatter diffraction (EBSD). The thermal decomposition of the samples was studied using a simultaneous thermogravimetric and differential thermal analyzer (DTG–60H, SHIMADZU corporation, Kyoto, Japan) under a N_2_ atmosphere at a rate of 5 °C/min, between 25 and 500 °C. To obtain elemental information, energy-dispersive X-ray fluorescence spectrometry (EDX8000, SHIMADZU corporation, Kyoto, Japan) was used. All the chemicals and reagents employed in these experiments were used as received without further purification.

### 2.2. Chemicals

Cellulose acetate (Mw = 10,000), poly sulfone (Mw = 10,000), hydrazinobenzoic acid (HBA), tetrahydrofuran (THF), dimethyl formamide (DMF), CuSO_4_, and NaCl were purchased from Sigma Aldrich (St. Louis, MO, USA).

### 2.3. Synthesis of the CA–HBA Modified Polymer

In a three-necked flask, 7 mmol. of an HBA solution in THF was added to 4 g of a CA solution. The reaction was allowed to react at 60 °C for 10 h under a nitrogen atmosphere. The product was precipitated in cold methanol (300 mL). The synthesized modified polymer was washed with hot methanol to dissolve the unreacted monomer and was then left to dry at room temperature.

### 2.4. General Preparation of Stock Solution of Metal Ions

A Cu (II) stock solution (200 ppm) was diluted with deionized water to achieve the desired concentration. The commonly used concentration for a stock copper solution is 10 ppm [30].

### 2.5. Procedures

#### 2.5.1. Fabrication of Membrane

The membrane was fabricated using a phase inversion technique. The polymeric solution (13 wt. % in DMF) was prepared and cast using an automatic film applicator, using a casting knife with a 250 µm thickness, a speed of 4 rpm, and a temperature of 25 °C. The phase inversion occurred in a coagulation bath of distilled water by immersing the steel sheet until the membrane was separated. Finally, the membrane was allowed to dry in air.

#### 2.5.2. Surface Hydrophilicity and Contact Angle Measurement

The hydrophilicity of the prepared membrane surfaces was measured as a function of the contact angle measurements using a Ramé-Hart goniometer, Succasunna, NJ, USA. A drop of distilled water (2 µL) was added to the surface of the membrane (3 cm × 2 cm) using a micro syringe. The contact angle was measured by adding a drop of water at five different positions within 20 s on the membrane’s surface.

#### 2.5.3. Method of Metal Ion Removal

A total of 300 mg of the membrane was immersed in 50 mL of a buffer solution with a definite pH of 5.5–9 for an aqueous solution of Cu (II) ions (6, 8, or 10 ppm). The mixture was shaken using a shaker at a speed of 200 rpm, at room temperature, for various intervals (30 min, 60 min, or 120 min). The mixture was separated by filtration. The adsorption uptake was determined by calculating the difference in concentration (before and after the adsorption process) using ICP for a quantitative determination, as shown in Equation (1):

(1) $$q_{e}(mg/g)=\frac{\left(C_{i}-C_{e}\right)V}{W}$$

where C_i_ is the initial concentration (ppm), C_e_ is the concentration at equilibrium (ppm), W is the mass of the adsorbent (g), and V is the volume of the solution (L).

The adsorbed metal content inside the polymeric matrix was determined through a qualitative investigation using EDX and SEM/EDS.

#### 2.5.4. Water Flux

The water flux was calculated using the following formula (Equation (2)):

(2) $$J=\frac{\Delta V}{A∆t}$$

where ∆V (L), A (m^2^), and t (h) are the permeate volume, membrane area, and filtration time interval, respectively [31,32].

#### 2.5.5. Membrane Porosity and Pore Size

The membrane porosity (ε) was determined using the following formula (Equation (3)):

(3) $$\left(\varepsilon \right)=\frac{\left(m_{w}-m_{d}\right)}{A\times L\times \rho }$$

where A, L, and ρ are the membrane area, membrane thickness, and pure water density, respectively; (m_w_ − m_d_) represents the mass loss of the wet membrane after drying.

The pore radius (rm) was calculated according to the Guerout–Elford–Ferry equation (Equation (4)):

(4) $$rm=\frac{\sqrt{\left(2.9-1.75\varepsilon \right)\times 8\eta LQ}}{\varepsilon \times A\times ∆P}$$

where ε is the overall porosity, η is the water viscosity at 25 °C, L is the membrane thickness (m), Q is the volume of the permeate water per unit time (m^3^/s), A is the effective area of the membrane (m^2^), and ΔP is the operational pressure (Pascal) [33,34].

#### 2.5.6. Regeneration of Metal Ions

The reusable potential of the membrane was examined by treating the membranes with an HNO_3_ solution. For inorganic fouling, the Cu (II) loaded onto the PSF membrane was gently washed with the distilled water, immersed in a definite amount of a 0.1 M HNO_3_ aqueous solution, and stirred at 500 rpm for 2 h with a slight increase in temperature [35]. Nitric acid was used as the eluent for the regeneration. The copper cations were transformed into copper nitrate, which is soluble in water. After washing with distilled water and drying, the regenerated membrane was reused. The EDX of the membrane, after leaching out the cations, did not show a Cu cation peak.

#### 2.5.7. Preparation of the NaCl Solution

A 10,000 ppm stock solution of NaCl salt was prepared in deionized water for the investigation of the desalination process.

#### 2.5.8. Desalination Process

The desalination experiment was conducted by immersing 0.3 g of the membrane in 100 mL of the stock solution under shaking conditions for a particular time (1 h, 2 h, or 3 h) at 200 revs. min^–1^. After filtration, the membrane was analyzed using SEM/EDS to confirm the extraction of salt into the polymeric matrix. The removal percentage of NaCl was determined using the conductivity of both the feed and the permeate salt solution [36] according to the following equation (Equation (5)).

(5) $$Removal\%=\frac{\left({Cond.}_{f}-{Cond}_{p}\right)V}{{Cond.}_{f}}\times 100$$

## 3. Results

### 3.1. Spectroscopic Analysis

**Figure 1:**
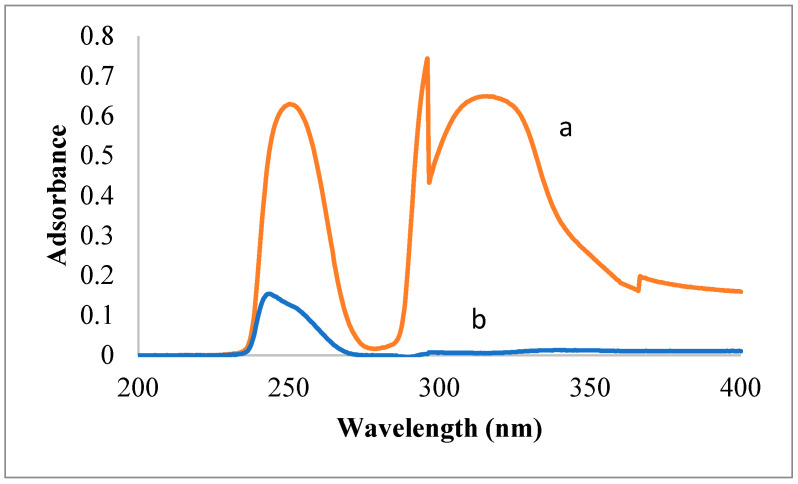
UV-Vis spectrum of CA-HBA (a) compared to CA (b).

The modification of cellulose acetate (CA) with hydrazinobenzoic acid (HBA) was performed with the aim of creating active sites in the polymeric matrix, such as carboxylic groups and basic nitrogen atoms. The modification process occurred in THF at 60 °C. The product was characterized using UV-Vis spectroscopy, as shown in Figure 1.

The modified polymer, CA-HBA, was characterized using a spectroscopic analysis. The UV-Vis spectrum of CA-HBA in Figure 1a shows an extra broad peak above λ = 300 nm compared to the CA spectrum in Figure 1b. This extra peak was derived from the HBA moieties inside the CA polymeric matrix [37]. To determine the modification of active reaction centers, the ^1^H NMR and ^13^C NMR spectra of the modified CA-HBA polymer were investigated.

**Figure 2:**
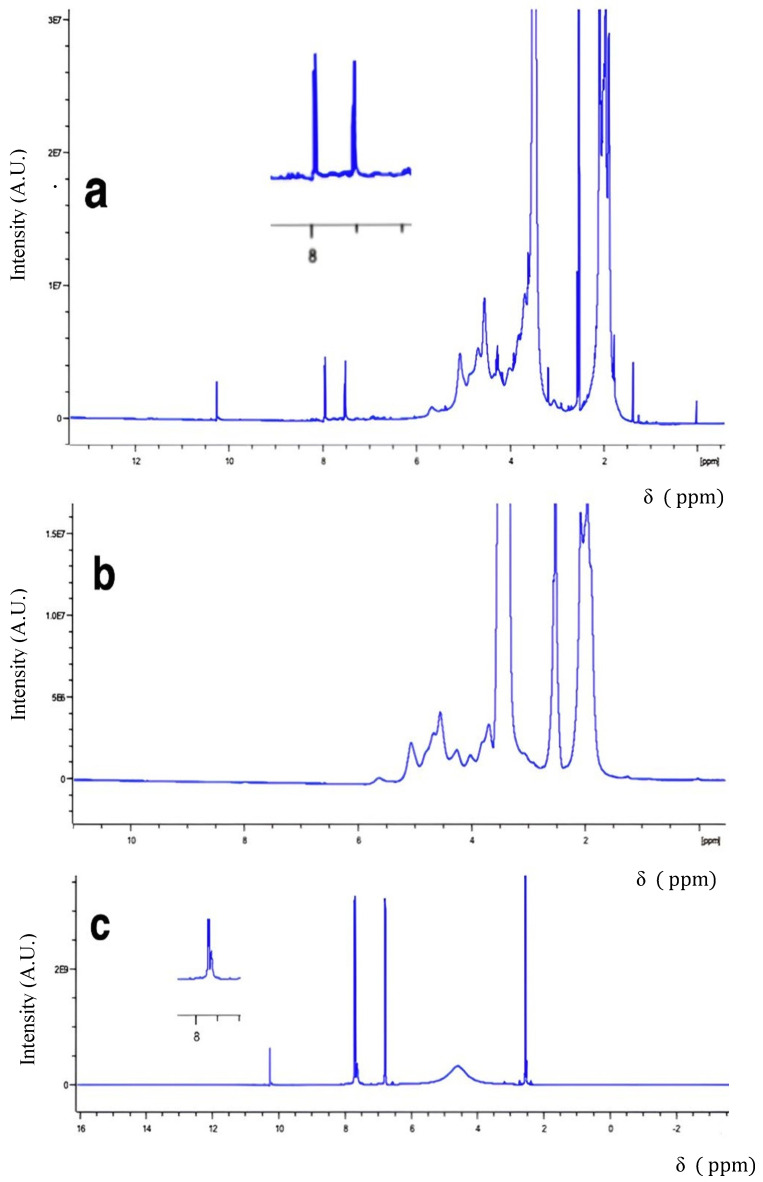
^1^H NMR spectrum of CA-HBA (**a**) compared to those of CA (**b**) and HBA (**c**).

**Scheme 1:**
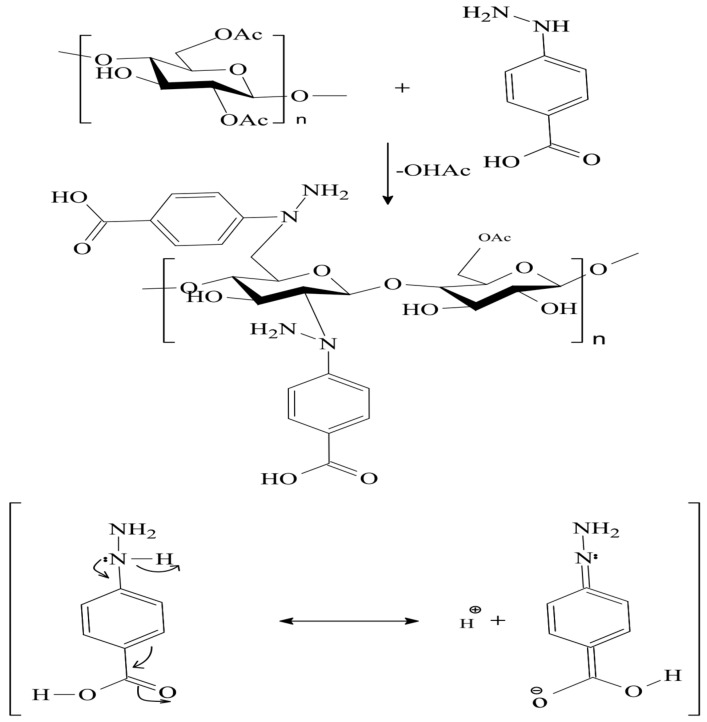
Modification reaction of CA with HBA.

Figure 2 shows the ^1^H NMR spectrum of CA-HBA (a) compared to those of CA (b) and HBA (c). The CA-HBA spectrum showed aromatic proton signals at 7.9 and 7.4 ppm. The presence of the carboxylic proton at 10.35 ppm confirmed that the carboxylic group was not consumed during the modification reaction. The -NH_2_ proton could not be identified, as it emerged with the signals related to CA between 4 and 5 ppm. The –NH proton (directly attached to the benzene ring) at 7.6 in the HBA spectrum disappeared in the spectrum of CA-HBA, indicating that the CA moiety reacted with HBA through the -NH group. The details of the ^1^H NMR spectral data are tabulated in Table 1. The reaction between CA and HBA is presented in Scheme 1.

**Figure 3:**
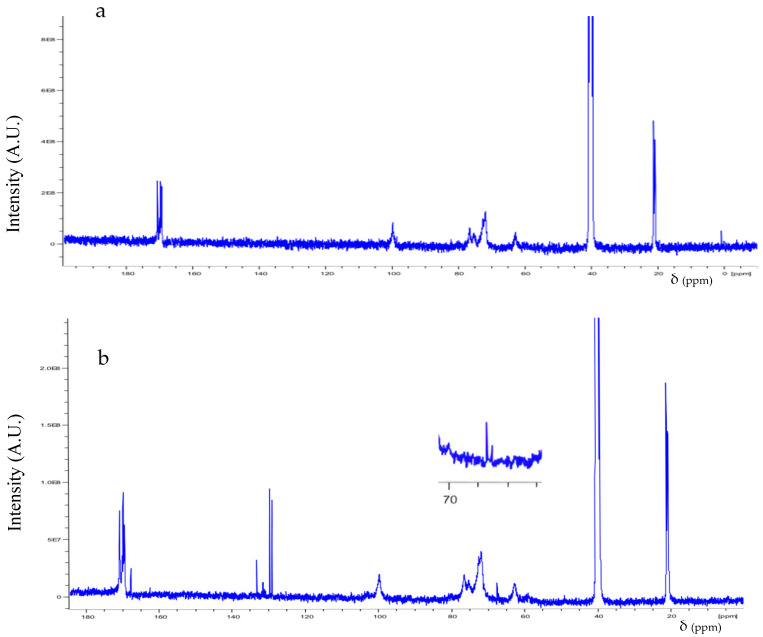
^13^C NMR spectra of CA (**a**), and CA-HBA (**b**).

For additional confirmation of the active reaction sites for the chemical modification of CA and HBA, ^13^C NMR was used (Figure 3). The ^13^C NMR spectrum of CA-HBA showed additional peaks compared to that for CA, according to the following carbon atoms: peaks at 168 ppm, corresponding to the carboxylic carbon C1; peaks at the region of 129–135 ppm, indicating the aromatic carbons (C2, C3, C4, and C5); and a distinguished peak at 67 ppm, indicating the methylene carbon where the modification took place at C6. Additionally, the peak corresponding to C7 of the glycosidic ring, where the modification with HBA also occurred, appeared at 67.5 ppm.

Thus, ^13^C NMR spectroscopy confirmed the active reaction sites of the modification process, and they coincided with the data from the literature [38,39]. The ^13^C NMR spectral data details are presented in Table 2.

### 3.2. Thermal Stability

**Figure 4:**
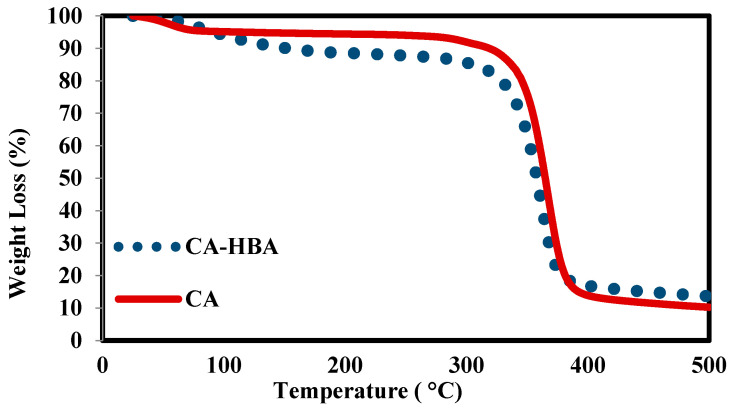
The TGA curve of CA-HBA (**. . . ** ) compared to CA (**^___^**).

Prior to suggesting that the CA-HBA polymeric material can be used in any water treatment applications, its thermal properties should be investigated. This was carried out using a TGA, as shown in Figure 4. The results revealed that the thermal stability of the modified polymer was comparable to that of CA. This was shown by the weight loss of CA-HBA, which was 86% at 500 °C, compared to CA, which is known to lose almost all its weight at 500 °C [90%]. The T° of CA-HBA showed a slightly lower value than that of CA due to the reduction in the crystallinity of the CA-HBA polymeric matrix compared to CA [38].

### 3.3. Crystallinity

**Figure 5:**
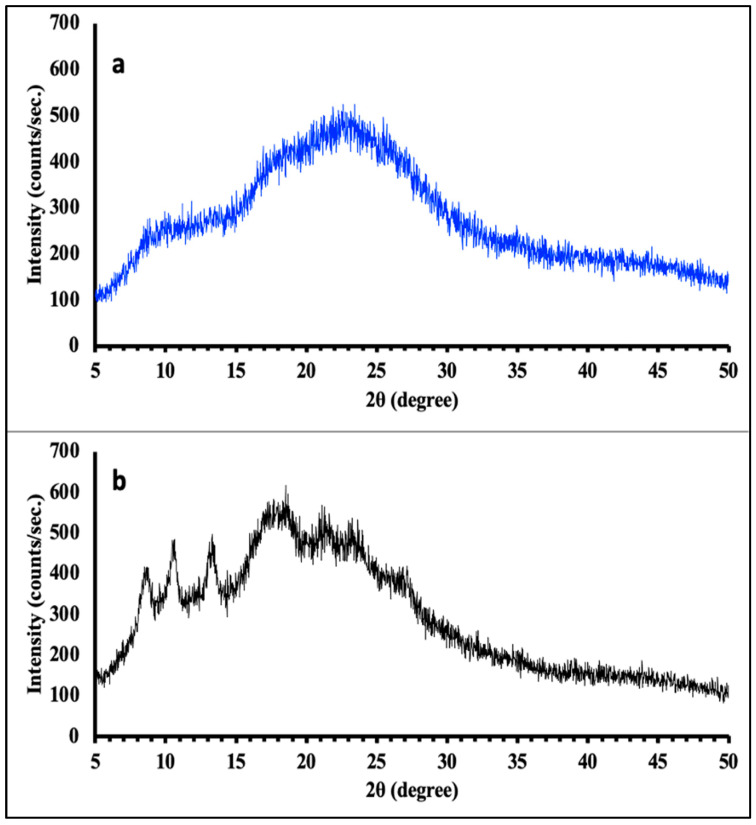
XRD patterns of CA (**b**), and CA-HBA (**a**).

The XRD pattern of CA showed sharp peaks at 2θ < 15, indicating the crystalline regions of the polymeric matrix, while the broad peak at 2θ > 17 corresponded to the amorphous region. The modified cellulose acetate showed only broad peaks, indicating a reduction in the crystallinity of the polymeric matrix (Figure 5). As a result of the substitution of the acetate groups by the large HBA moieties, the gaps between the main chains inside the polymeric matrix were increased, and this disrupted the formation of H-bonding through it.

### 3.4. Morphological Characterization

**Figure 6:**
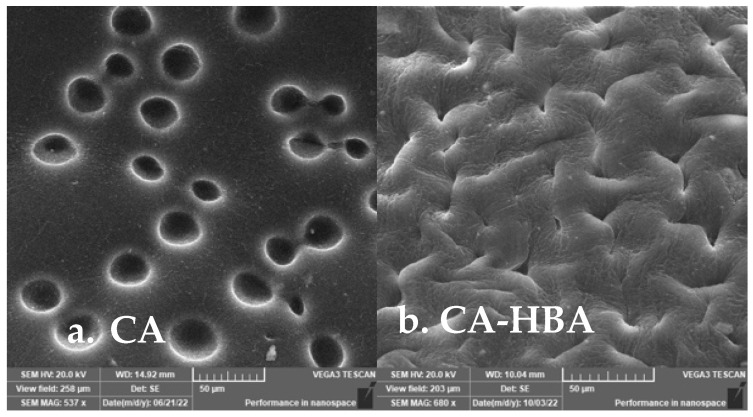
SEM images of CA (**a**) and CA-HBA (**b**).

The SEM/EDX technique was used to confirm the modified polymeric matrix. SEM showed a different morphology for the CA-HBA (Figure 6b) compared to that of CA (Figure 6a).

**Figure 7:**
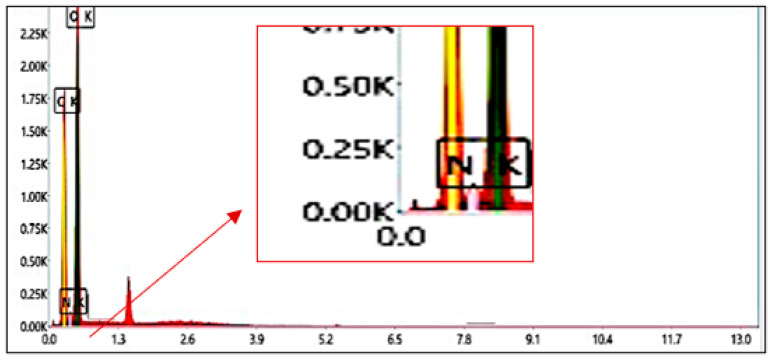
EDX of CA-HBA.

CA, as a polysaccharide, contains C, O, and H atoms only. The EDX of CA-HBA showed an extra peak corresponding to nitrogen atoms, which is a direct indication of the presence of HBA moieties inside the modified polymer (Figure 7).

**Figure 8:**
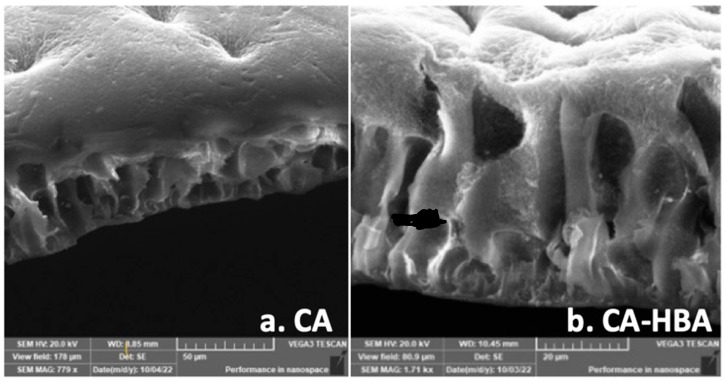
SEM images of the cross-sectional view of CA (**a**) and CA-HBA (**b**).

Moreover, an examination of a cross-section of the CA-HBA morphology showed a high porosity with finger-shaped pores, which would enhance the water flux and improve the membrane performance [35] (Figure 8).

**Figure 9:**
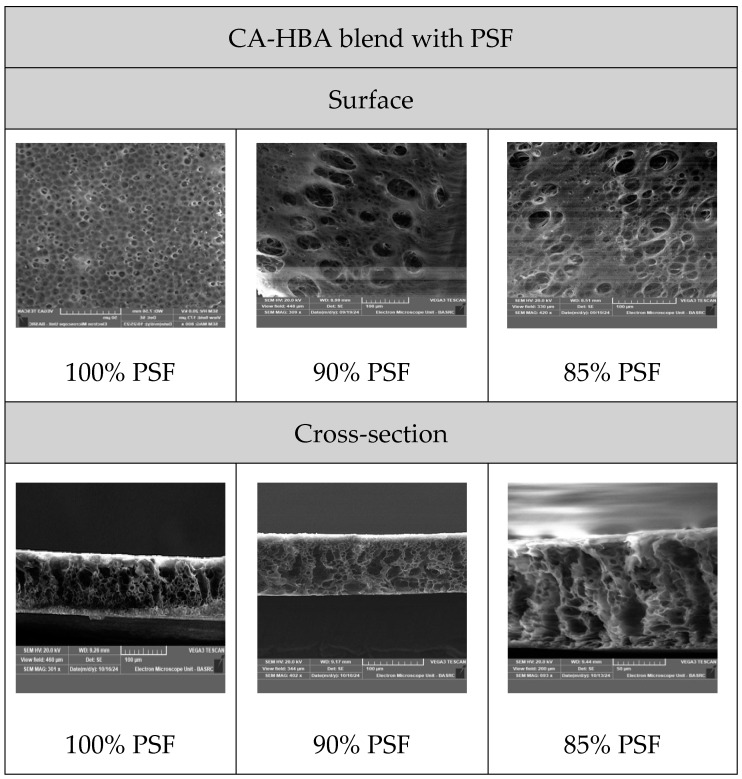
Surface and cross-section of CA-HBA/PSF blended membranes compared to those of PSF.

Thus, the modification of CA with HBA afforded the polymer with hydrophilic groups, which enhanced the water flux by creating finger-like pores through the polymeric membrane. However, it did not improve the membrane’s thermal stability. The polymer blend technique was chosen to enhance the thermal properties of the CA-HBA. Polysulfone (PSF) is known to exhibit a high thermal stability, as shown from its TGA data. It shows T° = 510 °C (Figure 9). Despite this advantage, PSF suffers from hydrophobicity due to its organic, nonpolar structure, which reduces the water flux and increases the water contact angle.

Although cellulose acetate and polysulfone are commonly used in the fabrication of membranes, both polymers should be modified prior to such fabrication using phase inversion techniques. Cellulose acetate exhibits the hydrophilicity needed for the water flux performance, while it suffers from poor heat stability at high temperatures. Polysulfone exhibits high thermal stability at high temperatures, but it is considered a hydrophobic polymer, which limits its adjustment to water treatment applications. In the present work, the polymer blend technique is suggested to integrate the benefits of both polymers for membrane manufacture.

### 3.5. Contact Angle and Surface Hydrophilicity

The CA membrane demonstrated moderate surface wettability (contact angle: 81°) due to the presence of hydroxyl groups. However, PSF exhibited a very low hydrophilic surface (contact angle: 94°). Blending PSF with CA-HBA led to an increase in the surface hydrophilicity of the resulting membrane. The hydrophilicity increased with an increase in the CA-HBA composition (Table 3). The membrane prepared from 15:85 CA-HBA:PSF exhibited the best surface hydrophilicity (contact angle: 69°) compared to the other membranes prepared from other blends and the parent PSF. This was attributed to the highest percentage of polar groups present in the HBA moieties [30].

### 3.6. Membrane Performance

To investigate the membrane’s efficiency for water treatment, some specific parameters should be examined, which include the water flux, porosity, pore size, and salt rejection.

#### 3.6.1. Water Flux

An area of the membrane (0.0051 m^2^) was kept in contact with water for a contact time of 1.5 h. Thus, the water flux would be affected by the quantity of water passing through the membrane over the given time (1.5 h). As hydrophilicity affects the water flux, increasing the percentage of CA-HBA in the polymer composition will enhance the water flux. The water flux increased from 9.8 (CA-HBA, 5%) to 30.2 (CA-HBA, 15%) (Table 4).

The surface of PSF showed a compact matrix and a sponge-like cross-section, which accounted for the low water flux of the pure PSF membrane. However, increasing the proportion of CA-HBA in the blend gave rise to a membrane with a cross-section showing finger-like channels, thus enhancing the water flux (Figure 9).

**Figure 10:**
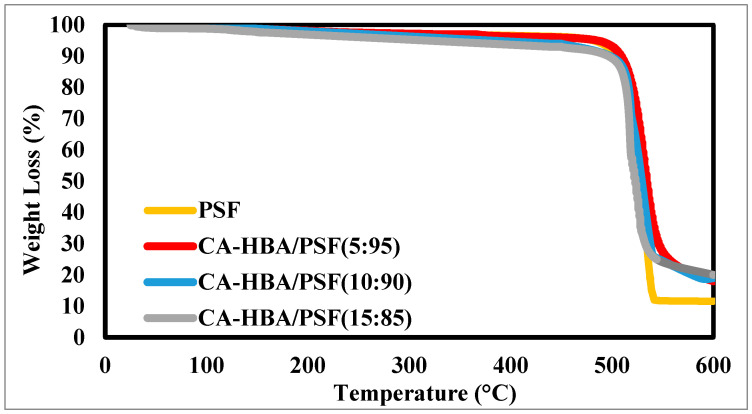
TGA curves of various CA-HBA/PSF membranes compared to PSF.

To determine the most suitable polymer blends for membrane manufacture, the thermal properties were examined using a TGA. The TGA data showed excellent thermal stability at high temperatures (up to 500 °C) for the polymer blend compositions with ≥85% PSF. The weight loss % was negligible (≤4%), as shown in Figure 10 and Table 5.

From the above-mentioned data for water flux and the TGA, three polymer blend compositions (PSF:CA-HBA, 95:5, 90:10, and 85:15) were chosen for the membrane fabrication.

#### 3.6.2. Membrane Porosity and Pore Size

The structure of a polymer affects the porosity percentage as well as the size of the pores. Table 6 shows the porosity percentage and pore size of various membranes fabricated from various blend compositions. The results revealed that both the porosity percentage and the pore size increased with an increase in the percentage of CA-HBA in the polymer membrane composition. This was attributed to the size and polarity of the carboxylic and amine groups, as polar groups increase the water content of the membrane [40].

#### 3.6.3. Removal of Cu (II) Ions

**Figure 11:**
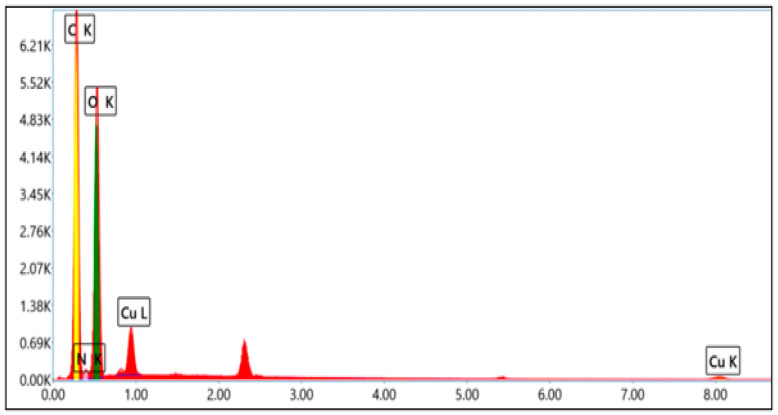
EDX of the 15:85 CA-HBA/PSF membrane after the removal of a 10 ppm Cu (II) solution.

The fabricated membranes (CA-HBA/PSF) exhibited high thermal stability, finger-like pores, and active adsorption sites (NH, COOH) for cations and salts. Thus, the membranes were expected to exhibit a good performance. The removal efficiency of the 15:85 CA-HBA/PSF membrane towards Cu (II) ions (10 ppm) at pH = 7 was investigated at room temperature for one hour. The EDX of the CA-HBA/PSF membranes showed two peaks corresponding to Cu cations, as shown in Figure 11.

### 3.7. Parameters Affecting Membrane Performance

#### 3.7.1. Contact Time

**Figure 12:**
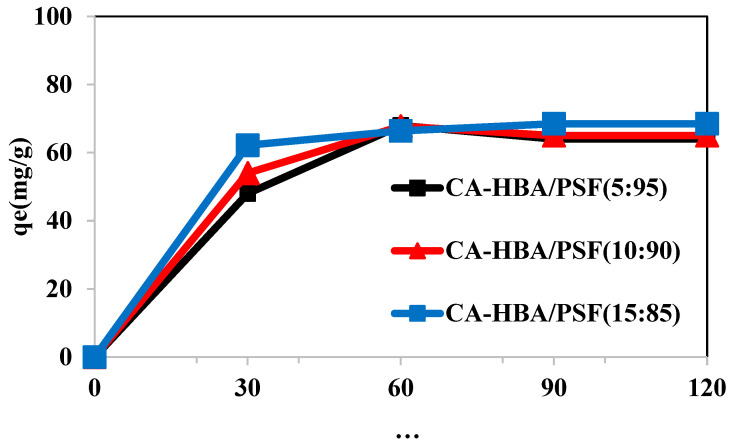
Effect of contact time on the removal efficiency of various membranes.

The results shown in Figure 12 reveal a gradual increase in the removal uptake with time for one hour; after this, no additional increase in the removal efficiency was observed with an increase in time. This may be attributed to the saturation of the active sites by Cu (II) ions [30].

#### 3.7.2. Effect of Adsorbate Concentration

**Figure 13:**
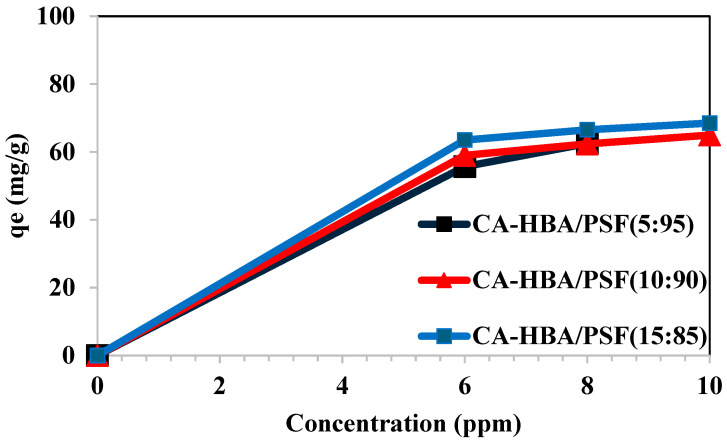
Effect of initial CuSO4 solution concentration on the removal process by various membranes.

Figure 13 shows the effect of the initial CuSO_4_ solution concentration on the removal process. The results revealed an increase in the removal efficiency up to an adsorbate concentration of 6 ppm; afterward, a slight increase was observed.

#### 3.7.3. Effect of pH

**Figure 14:**
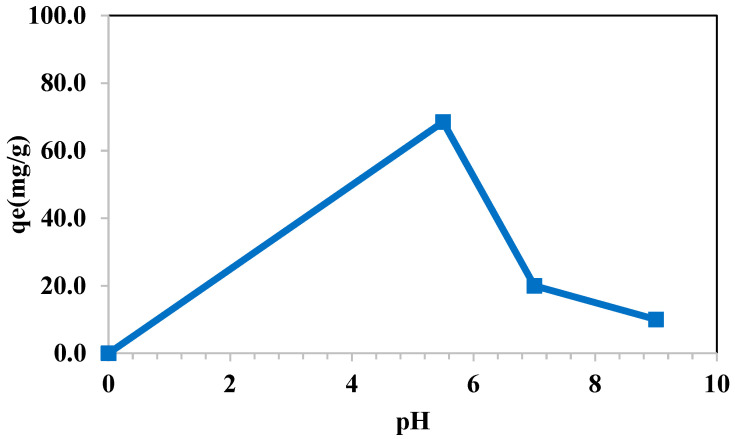
Effect of pH on the adsorption of Cu (II) by CA-HBA/PSF (15:85).

The most important factor affecting the cation removal process from aqueous solution is the pH of the solution. Figure 14 shows the removal efficiency of the membranes towards Cu (II) ions at various pH values, with all other factors being kept constant. The results reveal that the adsorption capacity reached its maximum value at pH = 7. Acidic pH values afforded the aqueous copper solution with extra protons, which competed with Cu (II) cations on the adsorption active sites, thus reducing the removal efficiency for the Cu (II) cations. However, basic pH values enhanced the precipitation of Cu(OH)_2_, thus decreasing the adsorption of the copper cations on the adsorption active sites [35,40].

From the above-mentioned data, it was determined that the optimal conditions for the removal of copper ions from aqueous solution by CA-HBA-PSF membranes are an absorbate concentration of 6 ppm, a contact time of one hour, and a pH = 7. Moreover, the 15:85 CA-HBA:PSF membrane exhibited the best copper cation uptake compared to the other blended membranes; this was attributed to the increase in the carboxylic groups of the HBA moieties in the polymeric matrix of this membrane. The carboxylic groups might have formed coordination or covalent bonds with the copper ions [40].

### 3.8. Desorption of Metal Ions and Reusability of the CA-HBA/PSF Membranes

**Figure 15:**
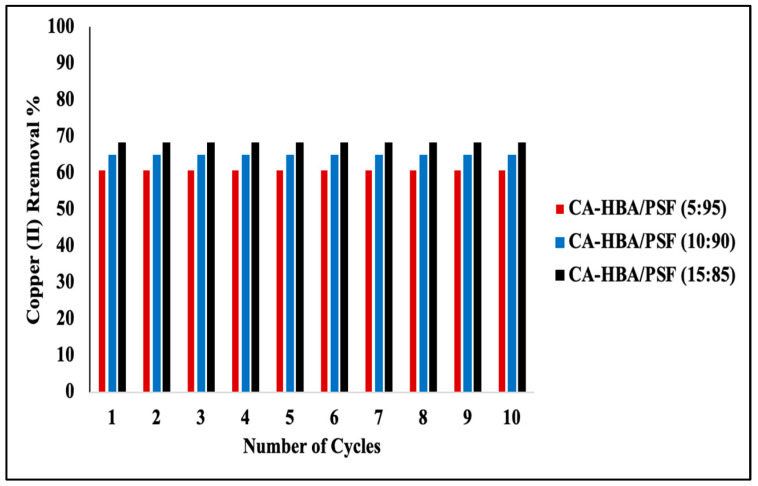
Removal percentages of various reused membranes without losing their efficiencies.

The desorption process occurred using a 0.1 M HNO_3_ solution. The volume of nitric acid affected the recovery %. The results revealed that a quantitative recovery was obtained for 10 mL of HNO_3_ (99%). All the membranes showed reusability up to 10 times without losing efficiency (Figure 15).

### 3.9. Desalination

The salt removal percentage was calculated from Equation (5). The results revealed that all the membranes exhibited excellent salt removal efficiencies compared to the low efficiency of PSF (Table 7).

The increase in HBA moieties corresponds to an increase in the number of carboxylic groups, which might have been transformed into sodium carboxylate [40].

**Figure 16:**
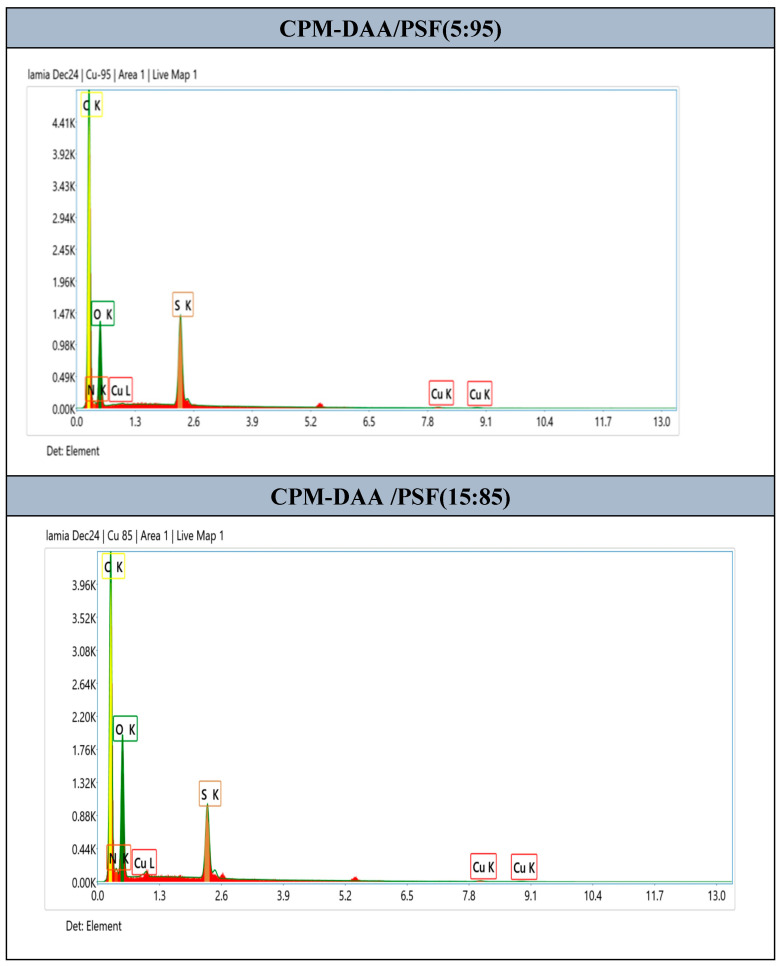
EDX of various CA-HBA/PSF membranes after the extraction of NaCl from a saline solution.

Figure 16 confirms the extraction of NaCl from water by various CA-HBA/PSF membranes using EDX. EDX showed the peak related to the sodium and chlorine atoms. Thus, the carboxylic groups acted as active removal sites for the extraction of Na^+^ cations.

**Scheme 2:**
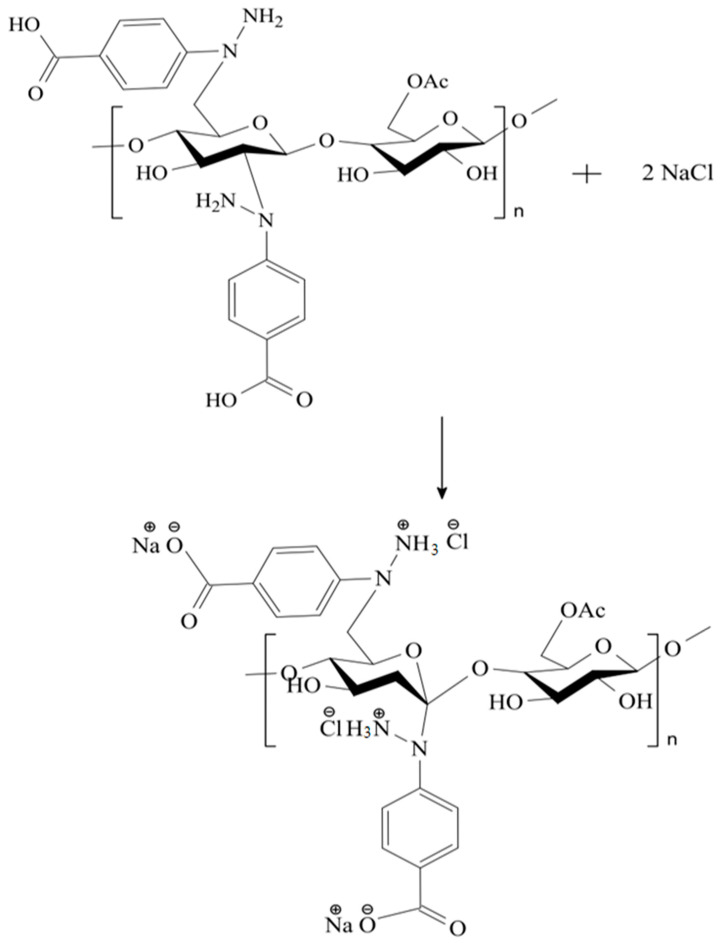
Schematic presentation of the trapping of NaCl inside the CA-HBA polymeric matrix.

**Figure 17:**
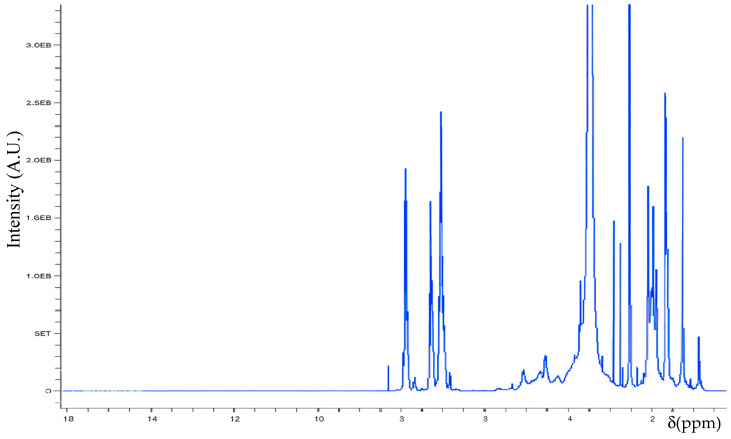
^1^H NMR of 5:95 CA-HBA/PSF blend after the extraction of NaCl salt.

Moreover, the extraction of NaCl by the 5:95 CA-HBA/PSF membrane was confirmed using ^1^H NMR spectroscopy after the desalination process (Figure 17). The peak related to the carboxylic proton at δ = 10.4 ppm disappeared as it was substituted with the sodium ions. Thus, the extraction of NaCl by the CA-HBA membranes occurred mainly through the trapping of salt inside the polymeric matrix, forming chemical bonds (–COONa), and not only through the influence of pressure and pore size (Scheme 2).

## 4. Conclusions

The modification of CA with HBA was characterized using SEM, ^1^H NMR, ^13^C NMR, and UV-Vis spectroscopy. Although the HBA moieties improved the hydrophilicity and increased the number of active sites for metal cation adsorption and salt extraction, they did not enhance the thermal stability at high temperatures. To address this, blending the modified polymer with PSF in different ratios was performed to improve the membrane’s thermal stability, as confirmed by the TGA data. Membranes with CA-HBA/PSF compositions of 15:85, 10:90, and 5:95 were selected for their superior thermal performance. Among these, the 15:85 CA-HBA/PSF membrane exhibited the highest removal efficiency for Cu (II) cations due to its higher content of carboxylic groups serving as active sites. Moreover, the 15:85 CA-HBA/PSF blend demonstrated the best desalination performance, achieving 89.8% salt rejection, which was attributed to the transformation of carboxylic groups present in the HBA moieties to the corresponding sodium carboxylates, as observed from the ^1^H NMR and EDX analyses. All the membranes showed reusability up to 10 times without losing efficiency.

## Figures and Tables

**Table 1:** ^1^H NMR spectral data of CA-HBA.

Structure	H Atom	δ (ppm)
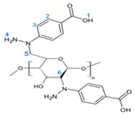	1	10.3
	2	7.9
	3	7.4
	4	4.3
	5	3.3
	6	3.1

**Table 2:** ^13^C NMR spectral data for CA-HBA.

Structure	C atom	δ (ppm)
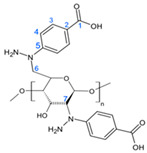	1	168
	2	130
	3	131.5
	4	129
	5	133.5
	6	67
	7	67.5

**Table 3:** Contact angle values of various membranes.

Membrane	Contact Angle	Image
PSF	94°	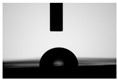
CA	81°	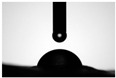
CA-HBA/PSF (5:95)	87°	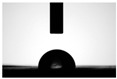
CA-HBA/PSF (10:90)	78°	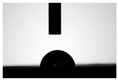
CA-HBA/PSF (15:85)	69°	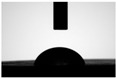

**Table 4:** Water flux of various CA-HBA/PSF membranes.

Membrane Composition	Water Flux (L/m^2.^h)
85% CA-HBA	30.2
90% CA-HBA	19.6
95% CA-HBA	9.8
100% PSF	0.1

**Table 5:** Thermogravimetric data for various membranes.

Membrane Composition	T° (°C)	Weight Loss% at 500 °C
100% CA-HBA	330	87%
15% CA-HBA	500	4%
10% CA-HBA	509	0%
5% CA-HBA	510	0%
100% PSF	510	0%

**Table 6:** Porosity percentage and pores size of various membrane.

Membrane Composition	Porosity %	Pore Size (µm)	Water Content
CA-HBA	72	0.192	86.8
PSF 85%	58	0.139	70.8
PSF 90%	53	0.137	66.7
PSF 95%	50	0.071	50
PSF	32	0.047	54

**Table 7:** Desalination efficiencies of CA-HBA/PSF membranes compared to that of PSF.

Membrane	Removal %
PSF	8.2
CA-HBA/PSF 95%	52.5
CA-HBA/PSF 90%	66.6
CA-HBA/PSF 85%	89.8

## Data Availability

The raw data supporting the conclusions of this article will be made available by the authors on request.
